# Prolonged corrected QT interval and Torsades de pointes following electrical cardioversion of atrial fibrillation in a young woman

**DOI:** 10.1093/omcr/omaf295

**Published:** 2026-02-18

**Authors:** Hassan Elzain, Mohamed Elkalifa Elawad Elhassan, Karar Mahmoud Nadir Mohamed, Mohamed Adel Mostafa, Heba Fathy Ismail, Jasem H Redha, Anas Bedawi Babiker

**Affiliations:** Department of Cardiology, Sudan Medical Specialization Board, Hospital Street, Khartoum, Sudan; University of Khartoum, Faculty of Medicine, Cardiology, Qasr street, Khartoum, Sudan; University Hospitals of North Midlands NHS Trust, Cardiology, Stoke-on-Trent, England, ST4 6QG, United Kingdom; Department of Cardiology, Sudan Medical Specialization Board, Hospital Street, Khartoum, Sudan; Letterkenny University Hospital, Letterkenny, County Donegal, F92 FC93, Ireland; Kuwait Ministry of Health, Cardiology, Sulaibikhat, Al Asimah Governorate, 90000, Kuwait; Kuwait Ministry of Health, Cardiology, Sulaibikhat, Al Asimah Governorate, 90000, Kuwait; Kuwait Institute for Medical Specialization, Adult Cardiology Fellowship, Safat, Al Asimah Governorate, 13001, Kuwait; Kuwait Ministry of Health, Cardiology, Sulaibikhat, Al Asimah Governorate, 90000, Kuwait; Royal Care International Hospital, Cardiology, Khartoum, Burri, Sudan

**Keywords:** atrial fibrillation, electrical cardioversion, QT prolongation, torsades de pointes, polymorphic ventricular tachycardia

## Abstract

Electrical cardioversion is a widely used and generally safe procedure for restoring sinus rhythm in atrial fibrillation. However, it may rarely precipitate significant proarrhythmic complications. We report the case of a 35-year-old woman with a history of atrial septal defect closure who presented with persistent symptomatic atrial fibrillation. She underwent successful synchronized direct current cardioversion, but immediately developed marked prolongation of the corrected QT interval (>600 ms) followed by recurrent episodes of torsades de pointes, one requiring defibrillation. Intravenous magnesium was administered, but QT prolongation persisted in association with hypotension and bradycardia. Dopamine infusion was initiated to increase heart rate, which contributed to gradual QT normalization. The patient recovered fully without recurrence of arrhythmia. This case highlights a rare but potentially life-threatening complication of cardioversion and underscores the importance of vigilant post-procedural monitoring and timely intervention, even in patients without prior repolarization abnormalities.

## Introduction

Electrical cardioversion is a well-established and widely utilized intervention for restoring sinus rhythm in patients with atrial fibrillation. While considered safe and effective, the procedure is not entirely without risk. Rare but serious complications, including proarrhythmic events such as QT interval prolongation and Torsades de Pointes, have been documented even in patients without prior repolarization abnormalities [1]. Prompt recognition of such adverse effects is crucial, as they may result in life-threatening arrhythmias if not managed appropriately. This report presents a unique case of post-cardioversion QT prolongation and Torsades de Pointes in a young woman with atrial fibrillation, underscoring the importance of close ECG monitoring following cardioversion.

## Case report

**Figure 1:**
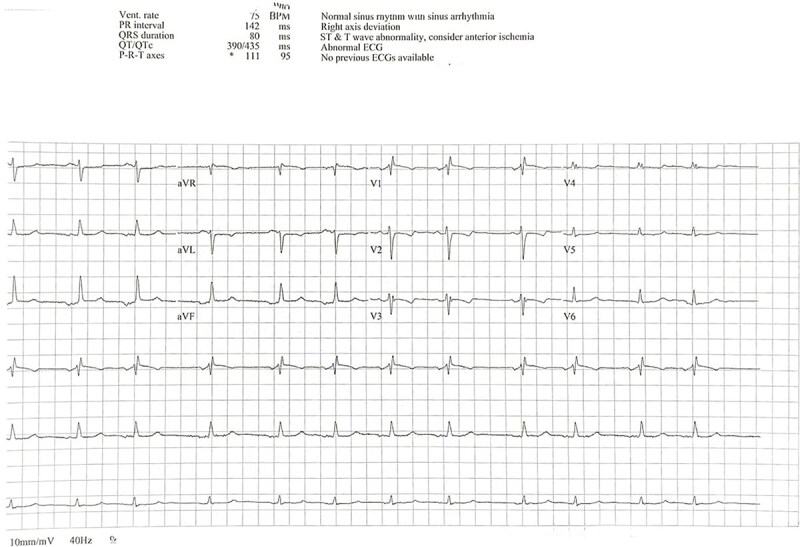
Baseline ECG showing normal sinus rhythm with normal QT interval.

A 35-year-old woman with a previous history of atrial septal defect (ASD) closure presented with symptomatic atrial fibrillation (AF). She had been diagnosed initially with paroxysmal AF in 2022, at which time her ECG showed sinus rhythm with a normal QT interval (Fig. 1). Bisoprolol was initiated, but she was subsequently lost to follow-up.

**Figure 2:**
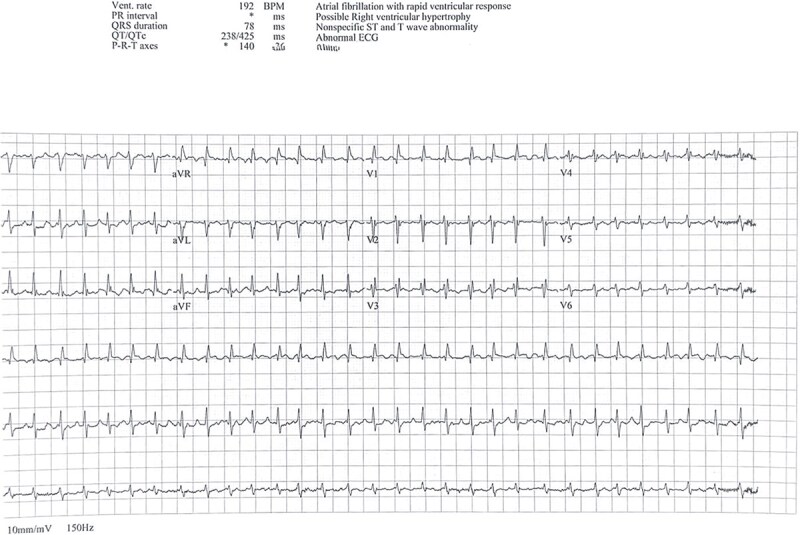
ECG showing atrial fibrillation prior to cardioversion.

From early 2025, she started experiencing increasingly frequent episodes of palpitations and fatigue. During the three months prior to presentation, her symptoms became persistent, and ECG confirmed progression to permanent AF (Fig. 2). She was initiated on apixaban 5 mg twice daily in addition to her ongoing bisoprolol. A transthoracic echocardiogram revealed mild left atrial dilation, mild mitral regurgitation, and preserved left ventricular systolic function without other significant abnormalities. Given her persistent symptoms, elective electrical cardioversion was planned.

**Figure 3:**
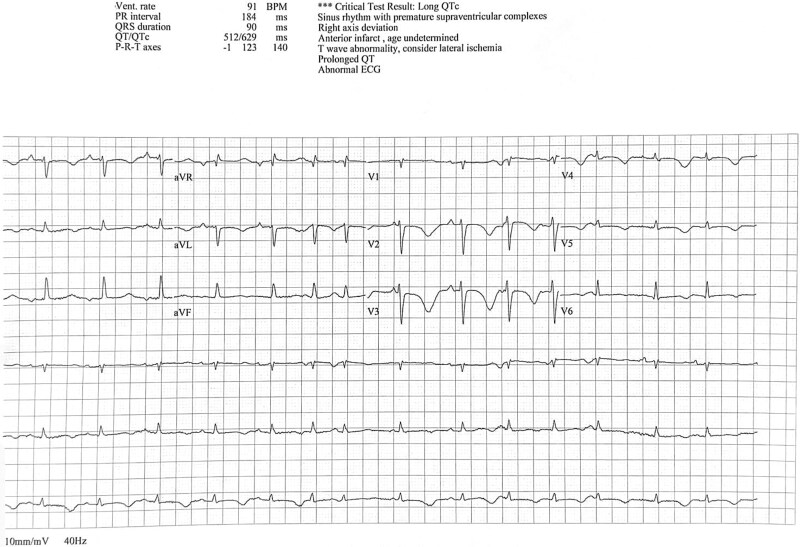
ECG immediately after cardioversion showing marked QT prolongation.

**Figure 4:**
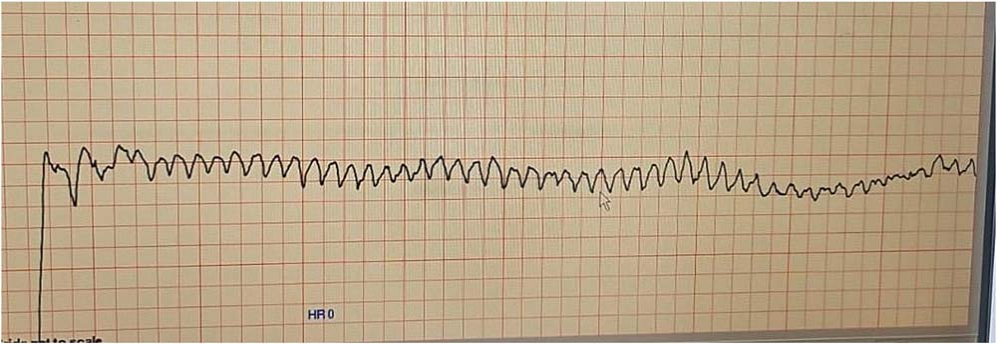
Telemetry recording of torsades de pointes following cardioversion.

The patient underwent synchronized direct current cardioversion (DCCV) under sedation, successfully restoring sinus rhythm. Immediately following the procedure, a repeat ECG revealed profound QT interval prolongation (QTc > 600 ms) (Fig. 3). Within minutes, she developed recurrent episodes of polymorphic ventricular tachycardia (Torsades de Pointes), documented clearly on telemetry monitoring (Fig. 4). One sustained episode necessitated immediate defibrillation, successfully restoring sinus rhythm.

In response, she was promptly managed with continuous intravenous magnesium infusion. Despite optimal magnesium supplementation, significant QT prolongation persisted, accompanied by hypotension (BP 90/50 mmHg) and relative bradycardia (heart rate approximately 60 bpm). Given the ongoing risk and the known association between bradycardia and QT prolongation, a dopamine infusion was initiated to elevate the heart rate and reduce the QT interval.

**Figure 5:**
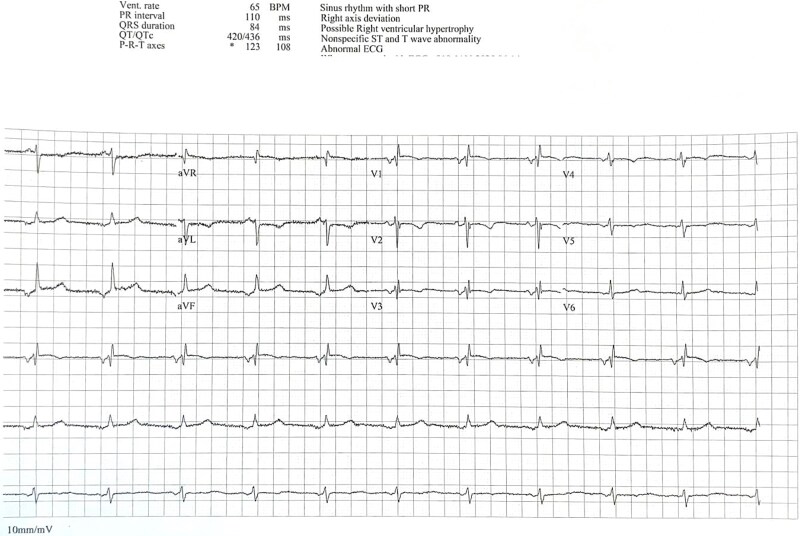
ECG on day 3 showing normalization of QT interval.

Over the subsequent 48 hours, there was gradual shortening of the QT interval. By the third day, repeat ECG confirmed normalization of QTc (Fig. 5). The patient remained hemodynamically stable thereafter, without recurrence of ventricular arrhythmias. Following normalization of the QT interval, genetic testing for congenital long QT syndrome was conducted and returned negative.

## Discussion

This case highlights an uncommon but potentially life-threat-ening complication following cardioversion of atrial fibrillation—marked QT prolongation leading to Torsades de Pointes. Although direct current cardioversion (DCCV) is generally safe and widely used to restore sinus rhythm, it carries a small risk of adverse events, particularly in the immediate post-procedural period [1].

Atrial fibrillation induces both electrical and structural remodeling in the atria and ventricles. Restoration of sinus rhythm through cardioversion can unmask transient repolarization abnormalities and lead to QT interval prolongation. Several mechanisms have been proposed to explain this phenomenon, including abrupt shifts in heart rate and autonomic tone, which may expose latent repolarization defects. These changes in ventricular repolarization dynamics are typically transient, but in rare instances, they can trigger malignant arrhythmias such as Torsades de Pointes [2, 3].

Our patient had a normal QT interval prior to cardioversion but developed a markedly prolonged QT interval immediately after restoration of sinus rhythm, eventually resulting in TdP. The absence of QT-prolonging medications, electrolyte abnormalities, or structural heart disease further supports a primary cardioversion-related mechanism.

QT prolongation and Torsades de Pointes (TdP) are medical emergencies that require immediate intervention. Intravenous magnesium sulfate is considered the first-line therapy for TdP, regardless of the serum magnesium level, due to its stabilizing effect on myocardial repolarization and ability to suppress early afterdepolarizations [4, 5]. In cases of hemodynamically unstable or sustained TdP, immediate direct current (DC) cardioversion is the treatment of choice. Delay in defibrillation may lead to degeneration into ventricular fibrillation and sudden cardiac death [6, 7]. In our patient, one sustained episode of TdP required prompt defibrillation, successfully restoring sinus rhythm.

In cases where QT prolongation persists despite magnesium therapy, especially when associated with bradycardia, increasing the heart rate becomes a critical strategy to prevent further episodes of TdP. Elevating the heart rate reduces the repolarization interval, thereby minimizing the risk of early afterdepolarizations that precipitate TdP. This can be achieved pharmacologically using agents such as isoproterenol or dopamine, or by temporary pacing in more severe or recurrent cases. In our patient, dopamine infusion was initiated to maintain a heart rate above 80 bpm, which contributed to QT interval normalization and arrhythmia suppression over the following days [7, 8].

This case underscores the critical need for vigilant ECG monitoring following electrical cardioversion, even in patients without prior repolarization abnormalities. While QT prolongation after cardioversion is often transient and benign, it may, in rare cases, precipitate life-threatening arrhythmias such as Torsades de Pointes. Early identification and timely intervention are essential, particularly when QT prolongation persists or arrhythmias recur despite appropriate therapy [7]. Although this is a single case and its findings cannot be generalized, it highlights a potentially underrecognized complication. Further studies are warranted to assess the prevalence, predictors, and optimal monitoring strategies for QT prolongation and arrhythmic events post-cardioversion.
